# Supplementary material for: Conserved perception of host and non-host signals via the a-pheromone receptor Ste3 in Colletotrichum graminicola
Source: Front Fungal Biol. 2024 Oct 7;5:1454633. doi: 10.3389/ffunb.2024.1454633 (PMC11491335; doi:10.3389/ffunb.2024.1454633)
Supplement: Supplementary file 1 [file DataSheet1.docx]

Supplementary Material

Conserved perception of host and non-host signals via the a-pheromone receptor Ste3 in *Colletotrichum graminicola*

Anina Yasmin Rudolph, Carolin Schunke, Daniela Elisabeth Nordzieke^*^

*** Correspondence:** Daniela Elisabeth Nordzieke: [dnordzi@gwdg.de](mailto:dnordzi@gwdg.de)

# Supplementary Figures and Tables

## Supplementary Figure

**
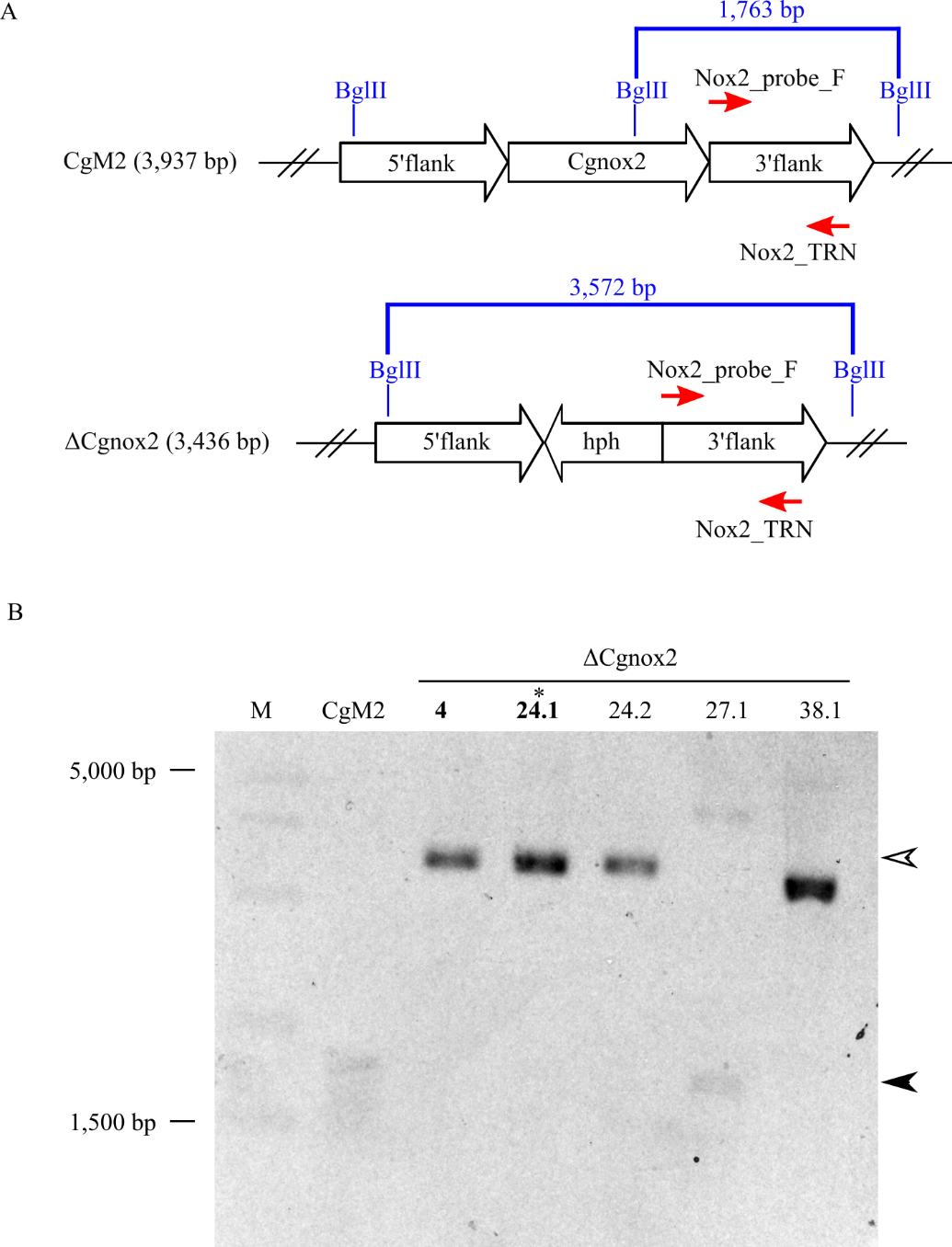
**

**Figure S1** Generation of a *Cgnox2* deletion strain in *C. graminicola*. **(A)** Depiction of the Southern blot strategy. Genomic loci of *Cgnox2* in the wildtype strain CgM2 and the deletion strain. Primer binding sites for amplification of the 3´flank region for the probe. Red arrows indicate the amplification direction. Recognition sites of restriction enzyme *Bgl*II and the resulting band sizes are indicated in blue. **(B)** Southern blot hybridization for verification of homologous integration of the hph-resistance cassette into the *Cgnox2* locus. The strains used for phenotypic characterization are written in bold letters. The single spore isolate T24.1 (asterisk) was used for complementation.

**
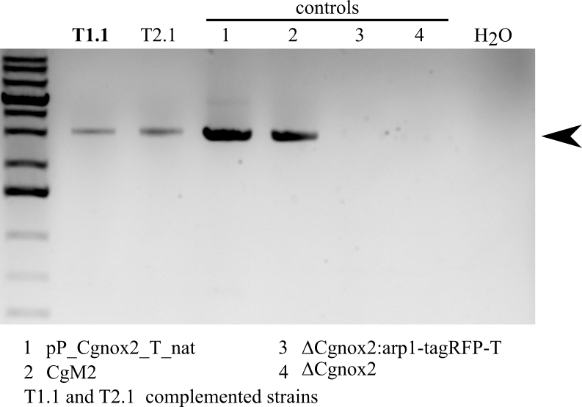
**

**Figure S2** Verification of ΔCgnox2::Cgnox2. Amplification with the oligonucleotides nox2_P_comp_fw and noxB_eGFP_YR_rv for verification of ΔCgnox2::Cgnox2 single spore isolates T1.1 and T2.1. The strain used for phenotypic characterization is written in bold letters. The correct band size of 2,892 base pairs is indicated with a black arrow. Positive controls 1 and 2 have the expected bands, while negative controls 3 and 4 show no amplification.


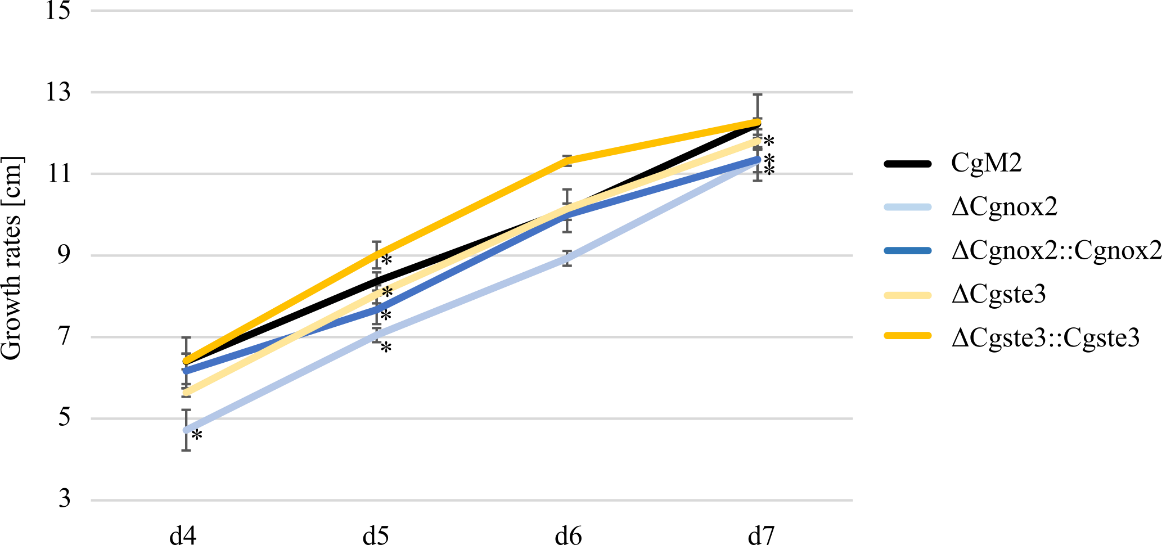


**Figure S3** Growth patterns of ΔCgste3 and ΔCgnox2. Growth areas were calculated in Fiji and growth rates were calculated from two subsequent days. n ≥ 3. * p < 0.05, calculated with two-tailed *t*-tests.

## Supplementary Tables

Table S1 Oligonucleotides used in this study

| Oligonucleotide | Sequence (5‘ to 3‘) |
| --- | --- |
| nox2_PF | CGAATACTCCGTACACCAAAAG |
| nox2_PR | TAGATGCCGACCGGGAACCAGTTAACGTGGTCTTCTTCTCGACTCTTTC |
| nox2_TF | AAAATGCTCCTTCAATATCAGTTAACTAAGTCAGCAAATTCGTTTCG |
| nox2_TR | AATATCGAACGGTTCTTGTACCTAG |
| hph-f | GTTAACTGATATTGAAGGAGCATTTTTGG |
| hph-r | GTTAACTGGTTCCCGGTCGGCATCTACTC |
| nox2_PFN | CGTCCTCTTCATGCTGAGAT |
| nox2_TRN | CTCTCAAGTCAAGGCGAATG |
| nox2_P_comp_fw | GATCTTCCGGATGGCGATATCCGTCCTCTTCATGCTGAGATCTC |
| nox2_T_comp_rv | ATGCCCTGCCCCTGAGATATCAATATCGAACGGTTCTTGTACCTAGC |
| Nox2_probe_F | GAAAGAAGCTGAGACGTGC |
| nox2_eGFP_YR_rv | GTGAACAGCTCCTCGCCCTTGCTCACCATGAAGTTCTCCTTGCCCCA |

Overhangs for the assembly reactions are indicated in red, inserted restriction sites for *Eco*RV are labelled in orange.

Table S2 *Colletotrichum graminicola* strains used in this study

| Strain | Genotype | Reference |
| --- | --- | --- |
| CgM2 (M1.001) | *C. graminicola* wild-type (wt) | (Forgey et al., 1978) |
| ΔCgnox2 | Homologous replacement of *Cgnox2* in CgM2, ssi, *hyg*^R^, *Cgnox2*::*hph* | This study |
| ΔCgnox2::Cgnox2 | Ectopic integration of pP_Cgnox2_T_nat in ∆Cgnox2, *nat*^R^, ssi; ∆*Cgnox2:: Cgnox2* | This study |
| ΔCgste3 | Homologous replacement of *Cgste3* in CgM2, ssi, *hyg*^R^, *Cgste3*::*hph* | (Rudolph et al., 2024) |
| ΔCgste3::Cgste3 | Ectopic integration of pCgste3_nat in ∆Cgste3, *nat*^R^, ssi; ∆*Cgste3*::*Cgste3* | (Rudolph et al., 2024) |
| ΔCgso | Homologous replacement of Cgso in CgM2, ssi, *hyg*^R^, *Cgso*::*hph* | (Nordzieke, 2022) |
| ΔCgso::Cgso | Ectopic integration of pCgso_c_nat in ∆Cgso, natR, ssi; ∆Cgso::Cgso | (Nordzieke, 2022) |

*nat^R^*: resistant to nourseothricin; *hyg^R^*: hygromycin resistant; ssi: single spore isolate, *hph*: hygromycin B phosphotransferase gene

Table S3 Plasmids used in this study

| Plasmid | Features | Reference |
| --- | --- | --- |
| pCgnox2_KO | *5‘ Cgnox2::hph::3’ Cgnox2, hyg^R^, amp*^R^ | This study |
| pCgnox2_nat | *5´ Cgnox2::Cgnox2::3´ Cgnox2*, *nat^R^, amp^R^* | This study |
| pJet1.2 | *amp^R^* | ThermoFisher Scientific |
| pJet_nat | *nat^R^, amp^R^* | (Nordzieke, 2022) |

*amp^R^*: ampicillin resistant; *gen^R^*: resistant to geniticin; *nat^R^*: resistant to nourseothricin; *gen^R^*; *URA3*: encodes for Orotidine-5'-phosphate (OMP) decarboxylase

**References**

Forgey, W., Blanco, M. and Loegering, W. (1978). Differences in pathological capabilities and host specificity of *Colletotrichum graminicola* on *Zea mays*. Plant dis. rep. *,* 62**,** 573-576.

Nordzieke, D. E. (2022). Hyphal Fusions Enable Efficient Nutrient Distribution in *Colletotrichum graminicola* Conidiation and Symptom Development on Maize. Microorganisms*,* 10**,** 1146.

Rudolph, A. Y., Schunke, C., Sasse, C., Antelo, L., Gerke, J., Braus, G., Poeggeler, S. and Nordzieke, D. E. (2024). Microbial two front attack: elaborate root infections by *Colletotrichum graminicola* oval conidia during maize anthracnose. bioRxiv**,** 2024.04. 05.588234.
